# Breast metastasis from colorectal cancer treated by multimodal therapy: Case report and literature review

**DOI:** 10.1097/MD.0000000000018016

**Published:** 2019-12-20

**Authors:** Tien-Chan Hsieh, Chao-Wen Hsu

**Affiliations:** a Division of Colorectal Surgery, Department of Surgery, Kaohsiung Veterans General Hospital, Kaohsiung City, Taiwan; b Department of Internal Medicine, Danbury Hospital, Danbury, CT; c Faculty of Medicine, National Yang-Ming University, Taipei City, Taiwan.

**Keywords:** breast metastasis, colorectal cancer, multimodal therapy

## Abstract

**Introduction:**

Breast metastases from primary colorectal carcinoma are extremely rare, with only 45 cases being reported previously. Since the most common malignancy in the breast and axilla is primary breast cancer regardless of cancer history, non-hematologic metastases may be misdiagnosed initially. Nevertheless, differentiating breast metastases from primary breast cancer is crucial because of their differences in prognosis and management.

**Patient concerns:**

We present a case of a 44-year-old Asian woman who noticed a new right breast lump after undergoing surgery and chemotherapy for her primary sigmoid colon cancer.

**Diagnosis:**

Image and immunohistochemistry findings were consistent with breast metastasis from primary colorectal adenocarcinoma.

**Interventions:**

The patient underwent breast tumor excision and reinitiated chemotherapy.

**Outcomes:**

The patient's disease progressed despite the interventions. She passed away 7 months after the detection of breast metastasis.

**Conclusion:**

When a new breast lesion is detected in patients with colorectal cancer history, the physician should consider the possibility of breast metastasis due to the poor prognosis. If a biopsy is necessary, cancer history should be provided to the clinicians to prevent incorrect pathological interpretation. In establishing the diagnosis, certain immunohistochemical markers have been shown to be sensitive and specific in previously reported cases. The combination of tumor excision and chemotherapy was the most common strategy in managing this condition with inconsistent clinical outcomes.

## 1 Introduction

There are ∼140,000 newly identified cases of colorectal cancer annually, and roughly 20% of them have distant metastasis at the initial presentation in the United States.^[1]^ Individuals with metastatic disease on presentation have worse prognosis.^[2]^ The regional lymph nodes, liver, lungs, and peritoneum are the most common metastatic sites.^[1]^ Breast metastasis of the primary colorectal cancer is extremely rare,^[3]^ and only 45 cases of breast metastases from the primary colorectal cancer have been identified in the literature.^[4–44]^ In fact, the most common breast metastatic source is from the contralateral primary breast cancer. Therefore, the diagnosis of primary or metastatic breast carcinoma is usually established for a newly identified mammary lesion despite patients’ prior cancer history. Nevertheless, differentiating the rare non-mammary breast metastases from primary cancer is crucial owing to their differences in management and prognosis.^[45]^ Here, we present a case of breast metastasis from sigmoidal adenocarcinoma in an Asian female treated with multimodal therapy.

## 2 Case report

**Figure 1:**
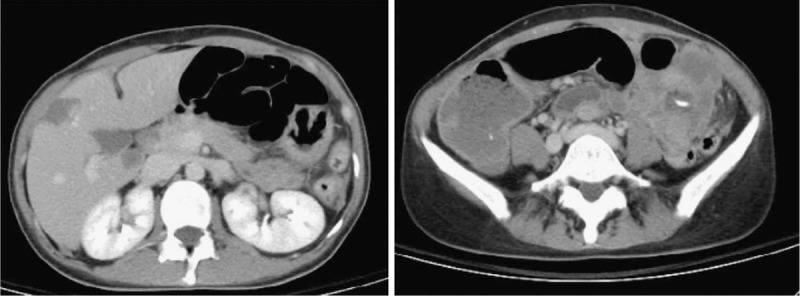
CT scan revealed 7 cm mass lesion of the sigmoid colon with visceral peritoneum and suspected pericolic/perirectal, superior rectal lymph node lesions. There was low grade bowel obstruction with fluid retention in the distal bowel loops. Bulky lymph nodes were noted over retroperitoneum, bilateral iliac chains, and right obturator region. Distant metastases were also suspected in hypodensity hepatic and splenic lesions.

**Figure 2:**
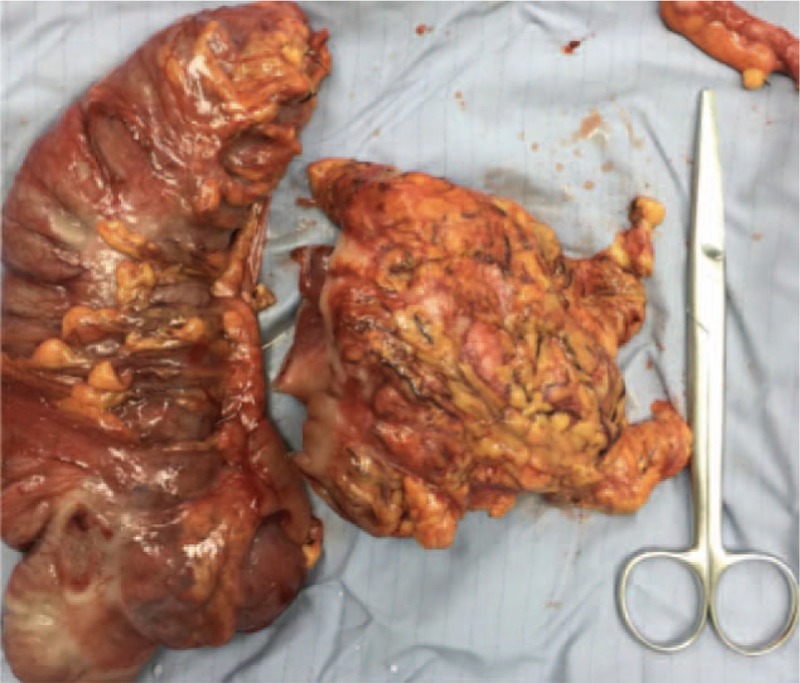
The gross specimen of sigmoidal tumor was measured 7 × 7 × 7 cm in size.

**Figure 3:**
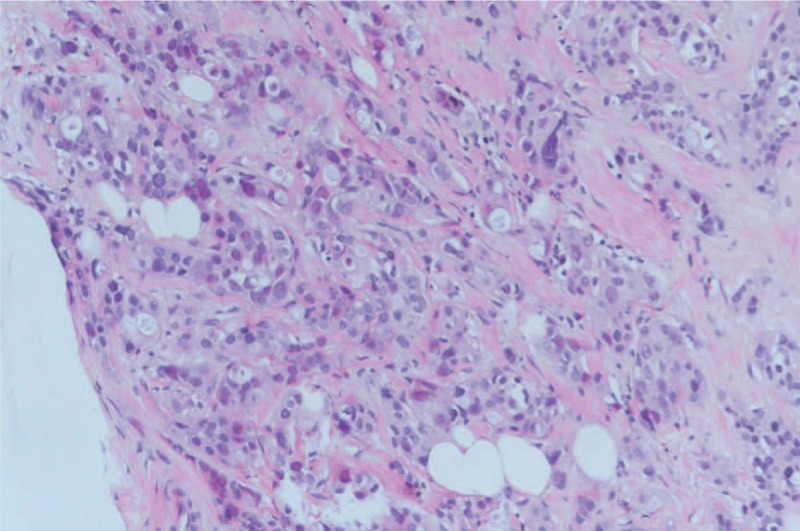
Microscopy examination of the debulking specimens were consistent with the diagnosis of adenocarcinoma with clusters of poorly differentiated neoplastic cells in solid and focal glandular pattern, desmoplastic stromal reaction, and tumor necrosis.

**Figure 4:**
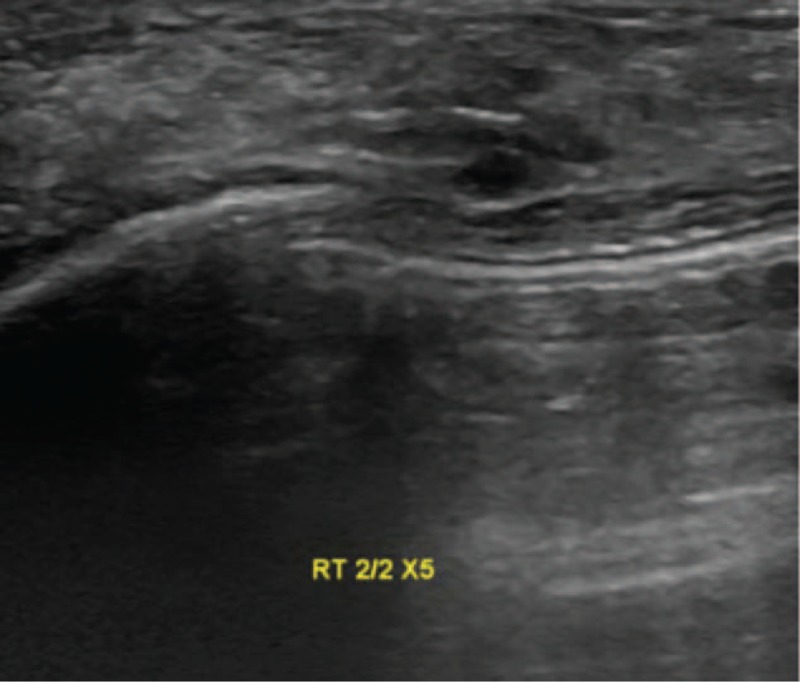
Ultrasound examination and percutaneous ultrasound-guided biopsy of the right breast lump.

A 44-year-old Asian woman had experienced intermittent abdominal pain, vomiting, and constipation for 3 weeks. Her symptoms persisted despite medical management. Physical examination revealed epigastric abdominal tenderness, abdominal guarding, and hypoactive bowel sounds. The initial computed tomography (CT) scan of the abdomen revealed an exophytic mass lesion measuring 7.0 cm at the sigmoid colon with visceral peritoneum invasion; 4 to 6 pericolic, superior rectal regional lymph node enlargement; bulky retroperitoneal lymph nodes; and multiple hepatic and splenic lesions (Fig. 1). The clinical stage was T4aN2aM1b (The Tumor, Node, Metastasis (TNM) staging system^[46]^). The specimen obtained from flexible sigmoidoscopy showed poorly differentiated adenocarcinoma. Due to bowel obstruction, the patient was admitted to undergo debulking operation and right colectomy; ileum and transverse colon segmental resection; and loop ileostomy with omental cake excision. The primary tumor measured 7 × 7 × 7 cm (Fig. 2). The pathological evaluation of the resected specimen was consistent with adenocarcinoma with clusters of poorly differentiated neoplastic cells in solid and focal glandular pattern with desmoplastic stromal reaction and tumor necrosis. Focal intracytoplasmic mucin was identified with mucicarmine staining (Fig. 3). Immunostaining showed positive result for cadherin 17; focally positive results for caudal-related homeobox gene 2 (CDX2), cytokeratin 20 (CK20), and synaptophysin; and negative results for cytokeratin 7 (CK7), neural cell adhesion molecule (CD56), and paired box 8, which were consistent with primary colorectal adenocarcinoma. *KRAS*, *NRAS*, and *BRAF* gene mutations were not detected. She was administered five cycles of FOLFIRI (Leucovorin Calcium (Folinic Acid), Fluorouracil, Irinotecan Hydrochloride) plus bevacizumab, four cycles of FOLFOX (Leucovorin Calcium (Folinic Acid), Fluorouracil, Oxaliplatin) plus panitumumab, and five cycles of FOLFOX plus bevacizumab. The changes in chemotherapy regimens were mainly due to intolerable adverse effects. One round of radiotherapy was provided as well. Seven months after the diagnosis of colorectal adenocarcinoma, the patient reported a new right breast lump. A firm 2 cm mass was found in the upper inner quadrant of the right breast at the 2 o’clock position, 2 cm from the nipple. The ultrasound identified 2 cm hypoechoic mass with ill-defined margin (Fig. 4). Both carcinoembryonic antigen (CEA) and carbohydrate antigen 19–9 (CA19–9) levels increased; however, carbohydrate antigen 125 (CA-125) levels were lower than the value after the initial debulking operation (CEA from 26.6 to 32.4 ng/mL, CA19–9 from 526 to 1338 U/mL, CA-125 from 168.8 to 15.8 U/mL). The patient underwent core biopsy, and the histological evaluation of the specimen identified adenocarcinoma with poorly differentiated neoplastic cells in solid and focal cribriform pattern with desmoplastic stromal reaction, tumor necrosis, and brisk mitosis. Immunohistochemical staining showed positive results for CDX2, CK20, and special AT-rich sequence-binding protein 2 (SATB2) and negative results for CK7, GATA3 binding protein (GATA3), human epidermal growth factor receptor 2 (HER2/neu), and estrogen and progesterone receptors (Fig. 5  ). The immunophenotype and histopathology of the newly identified breast lesion were comparable to the previous pathological findings of the patient's colorectal adenocarcinoma. Subsequent follow-up CT scan revealed progressive disease with enlargement of the liver and spleen metastasis (Response evaluation criteria in solid tumors [RECIST] 1.1 criteria^[47]^). The largest hepatic lesion measured 1.6 cm (Fig. 6). She underwent excisional operation for the breast lesion and received adjuvant FOLFOX plus bevacizumab. Despite the standardized chemotherapy regimens, the follow-up CT scan (Fig. 7) 6 months after the detection of breast metastasis revealed the enlargement of the hepatic lesions measuring 4.0 cm (1.6 cm in prior CT scan), progressive lymphadenopathies, and new bilateral lung lesions. The CT finding met the definition of progressive disease according to the RECIST guideline 1.1.^[47]^ Unfortunately, the patient passed away 14 months after the initial diagnosis and 7 months after the discovery of breast metastasis.

**Figure 5:**
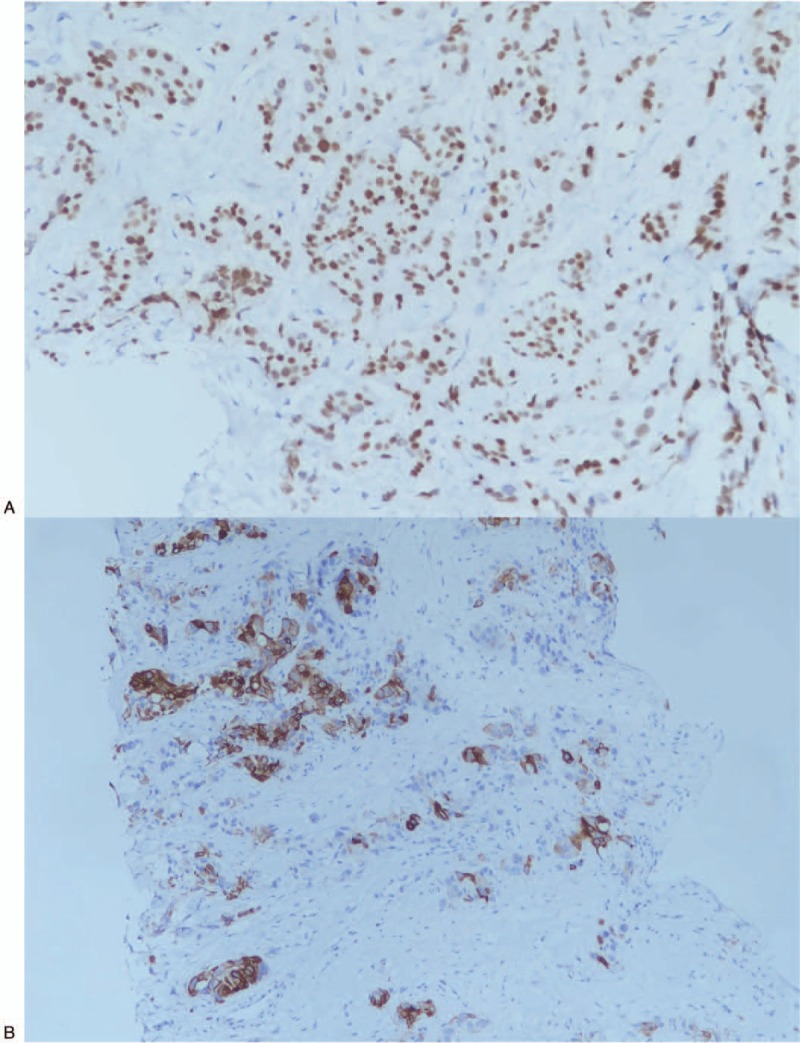
Immunohistochemical examination of the right breast biopsy specimen. (A) CDX2 positive. (B) CK20 positive. (C) SATB-2 positive. (D) CK7 negative. (E) Estrogen receptor negative. (F) GATA-3 negative.

**Figure 5 (Continued):**
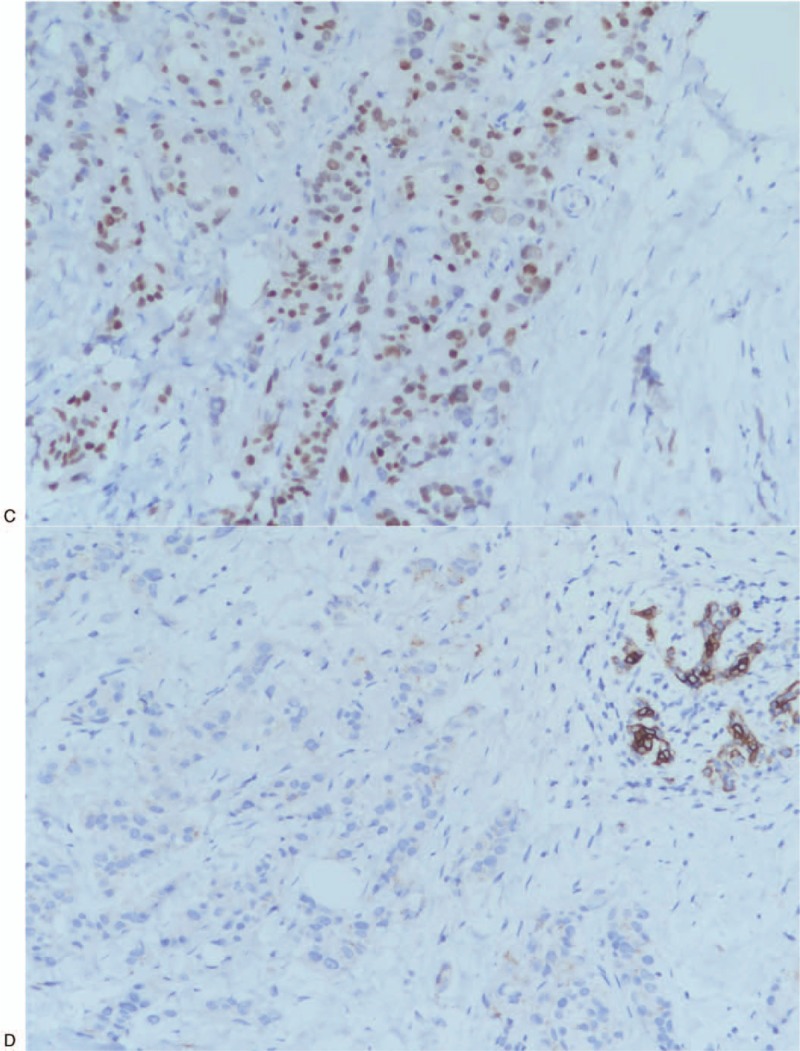
Immunohistochemical examination of the right breast biopsy specimen. (A) CDX2 positive. (B) CK20 positive. (C) SATB-2 positive. (D) CK7 negative. (E) Estrogen receptor negative. (F) GATA-3 negative.

**Figure 5 (Continued):**
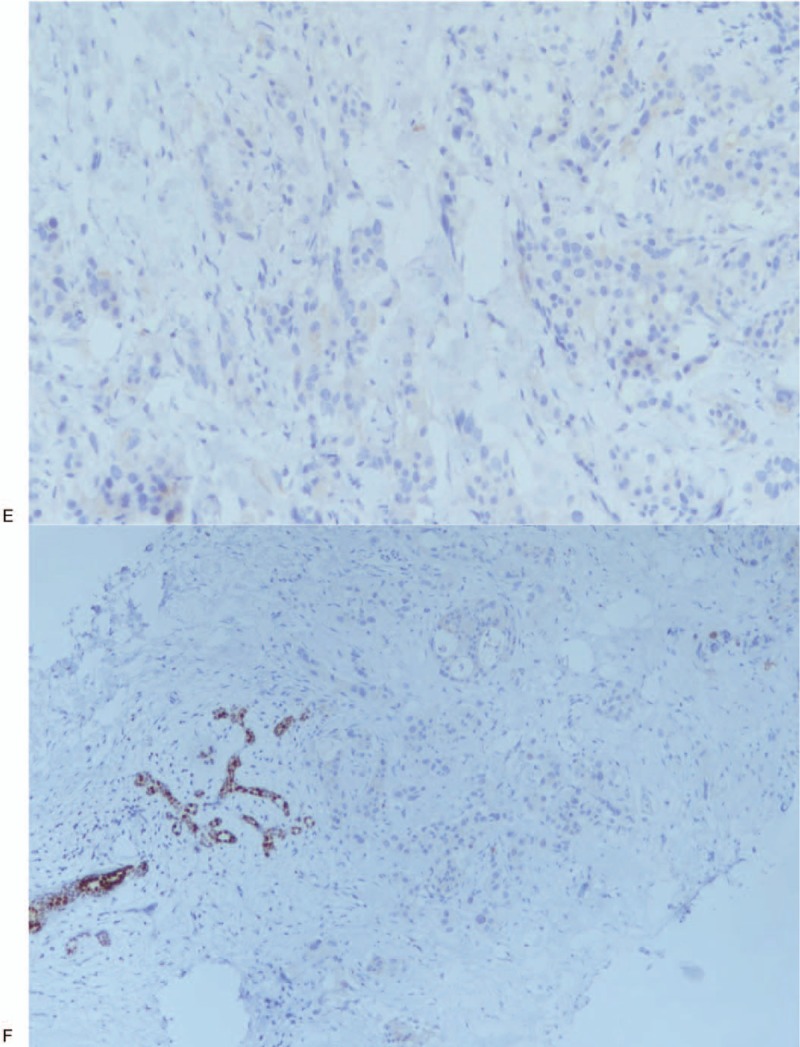
Immunohistochemical examination of the right breast biopsy specimen. (A) CDX2 positive. (B) CK20 positive. (C) SATB-2 positive. (D) CK7 negative. (E) Estrogen receptor negative. (F) GATA-3 negative.

**Figure 6:**
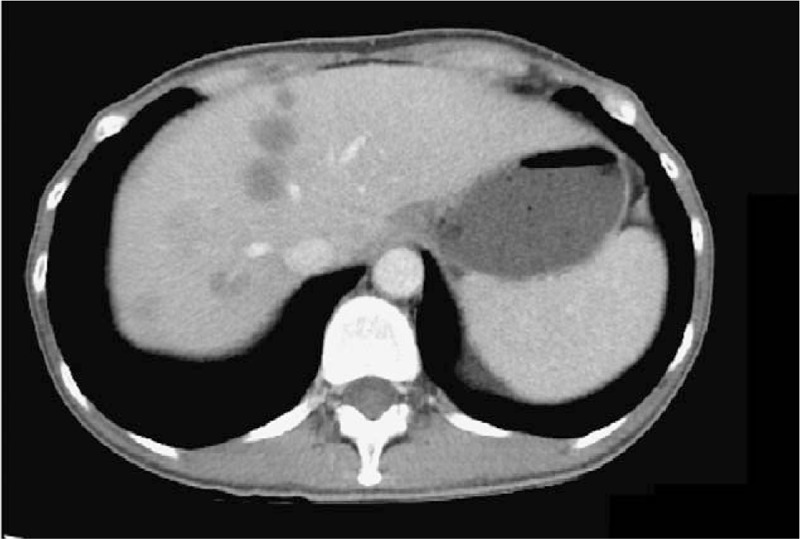
CT scan upon diagnosis of breast metastasis revealed disease progression (progressive disease by RECIST 1.1 criteria^[47]^). There were increased hepatic lesions, and the largest hepatic lesion was 1.6 cm.

**Figure 7:**
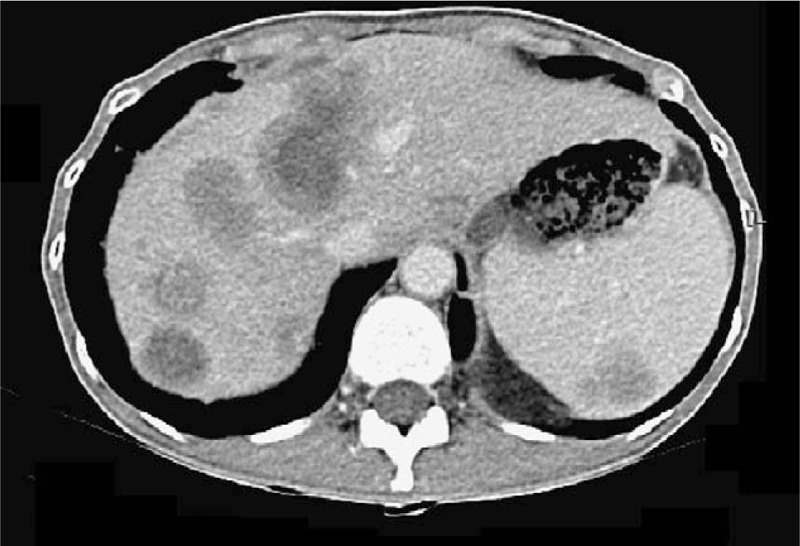
Follow-up CT scan 6 months after the diagnosis of breast metastasis showed evidence of progressive disease (RECIST 1.1 criteria^[47]^). The largest hepatic lesion was found 4.0 cm in size.

## 3 Discussion

Regardless of the patients’ cancer history, primary breast cancer is far more common than non-hematologic metastases to the breast and axilla. The most common sources of malignancies include the ovary, lung, skin, and stomach. However, colorectal carcinoma is among the rarest reported primary sites.^[42,45]^ The rarity may also lead to the initial misdiagnosis as physicians have insufficient knowledge regarding colorectal breast metastases. In fact, insufficient cancer history was reported to be a common reason for the incorrect pathological interpretation in a case series.^[45]^

We used PubMed and Google Scholar to identify any previous reports of breast metastasis of primary colorectal cancer. As a result, to the best of our knowledge, we summarized 45 cases that were previously reported in the literature along with our case in Table 1.^[4–44]^ The increasing number of identified cases can be due to the advancement of immunohistochemistry techniques.^[45]^

**Table 1:**
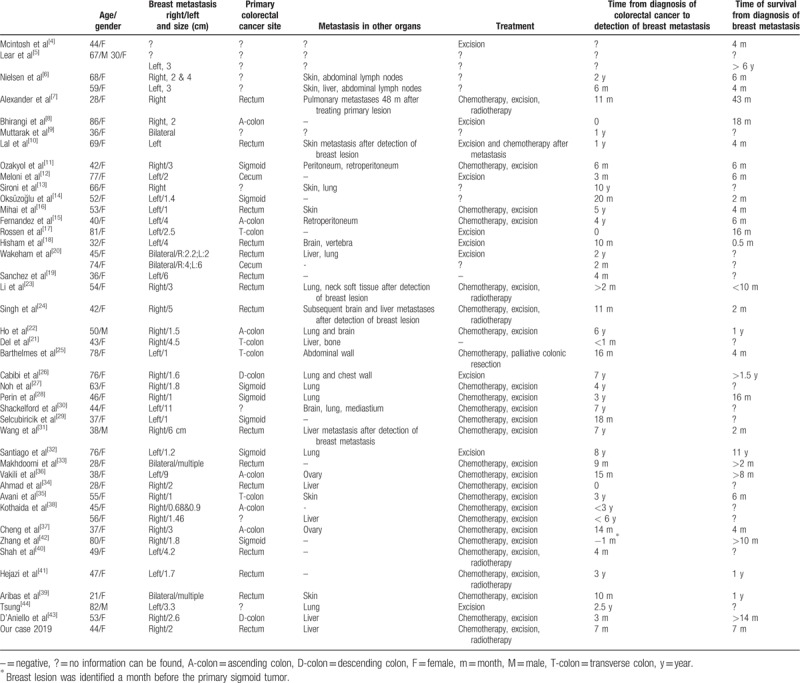
Summary of present case and all cases of breast metastases from primary colorectal cancer found in literature.

Recognition of breast metastases can be challenging since there is no reliable noninvasive diagnostic tool that detects breast metastases. In our case, the abnormal increase in CEA and CA19–9 levels resulted in the concern of disease progression. Unfortunately, the commonly used imaging studies for breast lesions, such as sonography and mammography, may not accurately distinguish between primary and secondary breast cancer. Although several imaging distinctions have been documented, nearly one-fourth of the breast metastasis cases were initially interpreted as benign or primary tumors in a case series.^[42,45]^ The specimen obtained from core biopsy of the breast is able to provide more diagnostic value. There are several histological features that can support the diagnosis of breast metastasis, such as a well-circumscribed lesion, lack of in situ ductal carcinoma and calcification, sharp transition to the adjacent tissue, and periodical and perilobular location.^[42,45]^ In previously identified cases, immunohistochemical studies were crucial in confirming the tumor origin and histological type, specifically for problematic cases without distinguishing morphological features. Among all the important markers, the most widely recognized are CK7 and CK20. Tumors originating from colorectal carcinomas are often negative for CK7 and positive for CK20, whereas breast ductal adenocarcinomas have the opposite feature (CK7+, CK20−). Estrogen and progesterone receptors, Her2, gross cystic disease fluid protein-15, and GATA3 are often positive in primary breast cancer but not in colorectal cancer.^[48,49]^ The immunophenotype of our case is consistent with that of the previously identified breast metastases from primary colorectal cancers.^[42,45]^ Furthermore, our case also shows positive results for CDX2 and SATB2, which have been shown to be both sensitive and specific for the tumor of colorectal origin.^[50,51]^

In all 46 cases (Table 1), the mean age at which breast metastases are detected is 52 years old and the majority of cases included females. For all cases with documented primary cancer site, more cases originated on the left side of the colon (descending colon, 2; sigmoid colon, 7; rectum, 15). This was consistent with the general cancer incidence by large intestine sections. Regardless of the primary tumor origin sites, metastatic lesions did not favor either side of the breast (left, 18; right, 21; bilateral, 5). The average time from diagnosis of colorectal cancer to the detection of breast metastases is 28.3 months. In one case, metastatic breast tumor was detected 10 years after the initial colorectal cancer. For all cases with survival time noted, the mean survival time after the detection of breast metastases is 14.9 months, with only 2 of the 31 cases surviving more than 5 years. These patients’ prognosis was worse than the general colorectal cancer patients with distant metastases, with a 5-year survival rate of 13.8%.^[52]^ Prior to the detection of breast lesions, patients often had a history of distal metastases to the liver, lung, and skin and abdominal and retroperitoneal lymph nodes. Three cases were found to have synchronous breast metastases with other organ metastatic lesions. However, in a small portion of the reported cases (12 cases), breast was the only distal metastasis. Interestingly, Zhang et al reported a case in which the initial presentation was the breast lesion for a patient with the primary sigmoid tumor that was identified later.^[42]^

Due to the poor prognosis and highly aggressive nature of breast metastasis of colorectal cancer, optimally, the physicians should individualize the treatment plan regarding the patients’ general conditions and goal of care. Majority of the patients received standardized management of their primary colorectal cancer. Because of the recurrent adverse effects, our case received chemotherapy including either FOLFIRI or FOLFOX plus bevacizumab or panitumumab. Most reported cases underwent both debulking operation and chemotherapy to control the overall tumor load and disease progression with inconsistent clinical outcomes. Our patient restarted FOLFOX plus bevacizumab after the excision of the breast tumor.

In conclusion, colorectal cancer metastasis to the breast is a rare but important diagnosis due to its poor prognosis. Immunohistochemical staining of biopsy or surgical specimens were the most important tools to establish the diagnosis, and several markers have been demonstrated to be highly sensitive and specific. Comparing to other colorectal cancer patients with distal metastases, breast metastases were associated with even worse overall survival. There is no consensus of managing this condition, but chemotherapy, excision, and radiotherapy were performed previously. Early recognition of this disease can potentially optimize the treatment plan and goal of care in these patients.

## 4 Declaration of patient consent

We have obtained the appropriate patient consent form. In the form, the patient has given her consent for her images and other clinical information to be reported in the journal. The patient understands that her names and initials will not be published and due efforts will be made to conceal her identity, but anonymity cannot be guaranteed.

## Author contributions

**Supervision:** Chao-Wen Hsu.

**Validation:** Tien-Chan Hsieh, Chao-Wen Hsu.

**Writing – original draft:** Tien-Chan Hsieh.

**Writing – review & editing:** Tien-Chan Hsieh, Chao-Wen Hsu.

Chao-Wen Hsu orcid: 0000-0002-7314-8423.
